# [(1,2,5,6-η)-Cyclo­octa-1,5-diene](1-ethyl-3-isopropyl-1,3-imidazol-2-yl­idene)(tri­phenyl­phosphane)rhodium(I) tetra­fluorido­borate

**DOI:** 10.1107/S2414314621005976

**Published:** 2021-06-11

**Authors:** Jeffrey A. Rood, Chhatra B. Subedi, John P. Risell, Andrei V. Astashkin, Edward Rajaseelan

**Affiliations:** a Department of Chemistry and Biochemistry, School of Science, Elizabethtown College, One Alpha Drive, Elizabethtown, PA 17022, USA; b Department of Chemistry, Millersville University, Millersville, PA 17551, USA; c Department of Chemistry and Biochemistry, The University of Arizona, Tucson, AZ 85716, USA; Vienna University of Technology, Austria

**Keywords:** crystal structure, rhodium, *N*-heterocyclic carbenes, cationic complexes

## Abstract

A new *N*-heterocyclic cationic rhodium(I) complex with a tetra­fluorido­borate counter-anion, [Rh(C_8_H_14_N_2_)(C_8_H_12_)(C_18_H_15_P)]BF_4_, has been prepared and structurally characterized. The cationic complex exhibits a distorted square-planar environment around the rhodium(I) ion. Two connections are made from rhodium(I) to the carbon atom of an *N*-heterocylic carbene ligand and to the phospho­rus atom of a tri­phenyl­phosphane ligand. The remaining two coordination sites are made *via* a bidentate inter­action from the two olefinic bonds of cyclo­octa­diene to the rhodium(I) ion. The compound includes an out-sphere tetra­fluorido­borate counter-anion. Within the crystal of the compound exist several weak inter­molecular C—H⋯F inter­actions.

## Abstract 2

The cationic complex of the title compound contains an Rh^I^ atom with a pseudo-square planar coordination environment. It is ligated by an *N*-heterocyclic carbene, a triphenylphosphane, and a bidentate cylco­octa­diene ligand. Charge balance is achieved from an out-sphere tetra­fluorido­borate anion.

## Graphical abstract

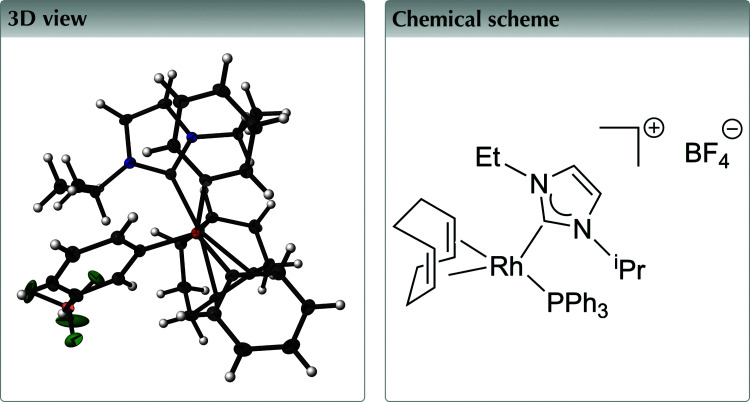

## Structure description

*N*-heterocyclic carbenes (NHCs) have emerged as excellent spectator ligands in homogeneous catalysis, especially in transfer hydrogenation reactions. Transfer hydrogenation of unsaturated bonds is a reaction of great inter­est and it exemplifies some of the key aspects of green chemistry (Ruff *et al.*, 2016 ▸; Zuo *et al.*, 2014 ▸). The *N*-heterocyclic carbene (NHC) ligands can be tuned sterically and electronically by having different alkyl groups on the nitro­gen atoms (Gusev, 2009 ▸). Many imidazole- and triazole-based NHC-rhodium and -iridium complexes have been synthesized and structurally characterized (Herrmann *et al.*, 2006 ▸; Wang & Lin 1998 ▸; Chianese *et al.*, 2004 ▸; Nichol *et al.*, 2009 ▸, 2010 ▸, 2011 ▸, 2012 ▸; Idrees *et al.*, 2017*a* ▸,*b* ▸; Huttenstine *et al.*, 2011 ▸). Their catalytic activities in the transfer hydrogenation of ketones and imines has also been studied and reported (Hillier *et al.*, 2001 ▸; Gnanamgari *et al.*, 2007 ▸; Albrecht *et al.*, 2002 ▸).

**Figure 1:**
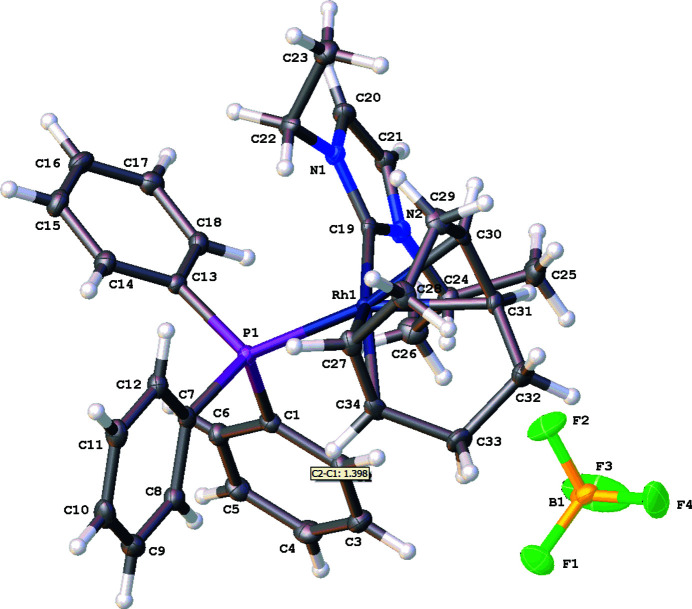
A view of the mol­ecular entities in compound (**4**), showing the atom labeling. Displacement ellipsoids are drawn at the 50% probability level.

The mol­ecular structure of the title salt, [RhC_34_H_41_N_2_P]^+^ (BF_4_)^−^, (**4**), is illustrated in Fig. 1 ▸. No solvent mol­ecules were found in the structure of (**4**). The coordination environment around the rhodium(I) ion, formed by the coordination to the metal of the two olefinic bonds of the cyclo­octa­diene (COD) ligand, the carbene carbon atom of the NHC ligand, and the phospho­rus atom from triphenylphosphane, is slightly distorted square-planar. The Rh—C(NHC) bond length is found to be 2.035 (3) Å in (**4**). The C(NHC)—metal—P(PPh_3_) bond angle is 88.37 (8)°. The N—C(carbene)—N bond angle in the imidazole-based carbene is 104.7 (2)°.

**Figure 2:**
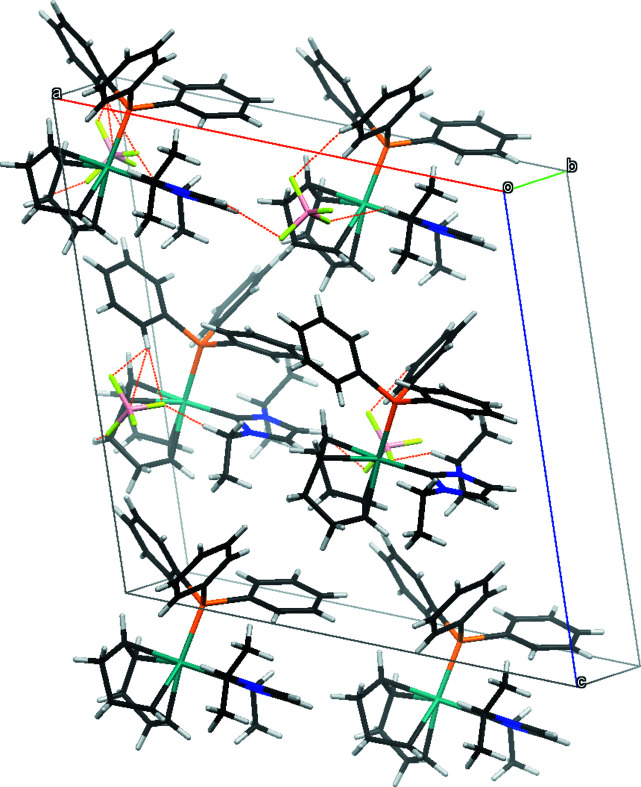
Crystal packing diagram of compound (**4**) with non-covalent inter­actions shown with dotted orange lines.

Several non-covalent inter­actions exist between atoms that are closer than the sum of the van der Waals radii and are reported in Table 1 ▸. Fig. 2 ▸ shows the crystal packing diagram for compound (**4**) with these inter­actions shown as dashed orange lines. The majority of these inter­actions exist as weak, unconventional C—H⋯F hydrogen bonds between the ligands and the fluorine atoms of the tetra­fluorido­borate anion. From the NHC ligand, the hydrogen atom on the five-membered ring, H21, inter­acts with F4. H24 from the isopropyl wingtip group and H22*A* from the ethyl wingtip group inter­act with F2 and F3, respectively. H28*B* and H32*B* from the double bonds of the COD ligand inter­act with F4 and F2, respectively. H12, a hydrogen atom in the *ortho* position of a phenyl ring on the tri­phenyl­phosphane ligand inter­acts with F1.

## Synthesis and crystallization

**Figure 3:**
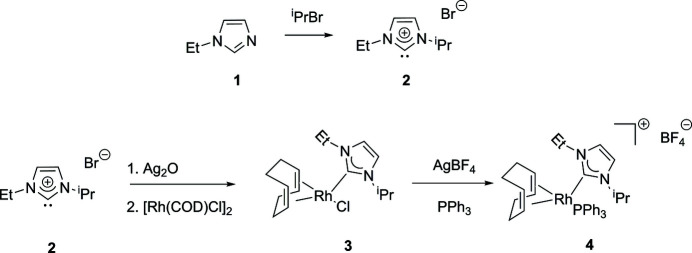
Reaction scheme summarizing the synthesis of the *N*-heterocylic carbene ligand through the formation of the title salt (**4**).

1-Ethyl imidazole (compound **1**) was purchased from Strem and used without further purification, and ligand syntheses were performed in air using reagent-grade solvents, which were used without further purification. NMR spectra were recorded at room temperature in CDCl_3_ on a 400 MHz (operating at 162 MHz for ^31^P) Varian spectrometer and referenced to the residual solvent peak (δ in ppm and *J* in Hz). A synthetic scheme is presented in Fig. 3 ▸. The imidazolium salt (**2**) was prepared by treating (**1**) with isopropyl bromide in toluene at reflux for 16 h followed by isolation with diethyl ether. The metal complex (**3**) was prepared by *in situ* transmetallation from silver carbene complexes of (**2**) (Chianese *et al.*, 2003 ▸). The title complex, (**4**), was prepared by treating (**3**) with 1 equivalent of tri­phenyl­phosphane and AgBF_4_ in CH_2_Cl_2_ at room temperature in the dark. The yellow–orange complex (**4**) was obtained in greater than 90% yield. ^13^C NMR: δ174.1(*d*, Rh—C, *J*(Rh—C) = 49.6). ^31^P NMR: δ25.48 (*d*, *J*(Rh—P) = 139.16). X-ray quality crystals of (**4**) were grown from CH_2_Cl_2_/pentane by slow diffusion.

## Refinement

Crystal data, data collection and structure refinement details are summarized in Table 2 ▸.

## Supplementary Material

Crystal structure: contains datablock(s) I. DOI: 10.1107/S2414314621005976/wm4147sup1.cif

Structure factors: contains datablock(s) I. DOI: 10.1107/S2414314621005976/wm4147Isup2.hkl

CCDC reference: 2088832

Additional supporting information:  crystallographic information; 3D view; checkCIF report

## Figures and Tables

**Table 1:** Hydrogen-bond geometry (Å, °)

*D*—H⋯*A*	*D*—H	H⋯*A*	*D*⋯*A*	*D*—H⋯*A*
C12—H12⋯F1^i^	0.95	2.57	3.245 (3)	129
C21—H21⋯F4^ii^	0.95	2.52	3.082 (3)	118
C22—H22*A*⋯F3^i^	0.99	2.39	3.120 (3)	130
C24—H24⋯F2	1.00	2.37	3.139 (3)	133
C32—H32*B*⋯F2	0.93 (3)	2.57 (3)	3.094 (3)	116 (2)
C28—H28*B*⋯F4^i^	0.98 (4)	2.53 (4)	3.318 (4)	138 (3)

Symmetry codes: (i) 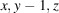 ; (ii) 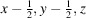 .

**Table 2:** Experimental details

Crystal data	
Chemical formula	[Rh(C_8_H_14_N_2_)(C_8_H_12_)(C_18_H15P)]BF_4_
*M*_r_	698.38
Crystal system, space group	Monoclinic, *C* *c*
Temperature (K)	100
*a*, *b*, *c* (Å)	17.4184 (15), 10.2177 (8), 18.5136 (16)
β (°)	109.164 (3)
*V* (Å^3^)	3112.4 (5)
*Z*	4
Radiation type	Mo *K*α
μ (mm^−1^)	0.65
Crystal size (mm)	0.40 × 0.26 × 0.09

Data collection	
Diffractometer	Bruker APEXII CCD
Absorption correction	Multi-scan (*SADABS*; Krause *et al.*, 2015 ▸)
*T*_min_, *T*_max_	0.672, 0.746
No. of measured, independent and observed [*I* > 2σ(*I*)] reflections	18836, 6836, 6747
*R*_int_	0.014
(sin θ/λ)_max_ (Å^−1^)	0.643

Refinement	
*R*[*F* ^2^ > 2σ(*F* ^2^)], *wR*(*F* ^2^), *S*	0.016, 0.041, 1.05
No. of reflections	6836
No. of parameters	407
No. of restraints	3
H-atom treatment	H atoms treated by a mixture of independent and constrained refinement
Δρ_max_, Δρ_min_ (e Å^−3^)	0.54, −0.29
Absolute structure	Flack *x* determined using 3291 quotients [(*I* ^+^)-(*I* ^-^)]/[(*I* ^+^)+(*I* ^-^)] (Parsons *et al.*, 2013 ▸).
Absolute structure parameter	−0.024 (5)

Computer programs: *APEX2* (Bruker, 2013 ▸), *SAINT* (Bruker, 2013 ▸), *SHELXT* (Sheldrick, 2015*a* ▸), *SHELXL* (Sheldrick, 2015*b* ▸), and *OLEX2* (Dolomanov *et al.*, 2009 ▸).

## full crystallographic data

### Crystal data

**Table:**

[Rh(C_8_H_14_N_2_)(C_8_H_12_)(C_18_H15P)]BF_4_	*F*(000) = 1440
*M**_r_* = 698.38	*D*_x_ = 1.490 Mg m^−^^3^
Monoclinic, *C**c*	Mo *K*α radiation, λ = 0.71073 Å
*a* = 17.4184 (15) Å	Cell parameters from 9851 reflections
*b* = 10.2177 (8) Å	θ = 2.4–27.2°
*c* = 18.5136 (16) Å	µ = 0.65 mm^−^^1^
β = 109.164 (3)°	*T* = 100 K
*V* = 3112.4 (5) Å^3^	Plate, yellow–orange
*Z* = 4	0.40 × 0.26 × 0.09 mm

### Data collection

**Table:**

Bruker APEXII CCD diffractometer	6836 independent reflections
Radiation source: sealed tube	6747 reflections with *I* > 2σ(*I*)
Graphite monochromator	*R*_int_ = 0.014
Detector resolution: 8 pixels mm^-1^	θ_max_ = 27.2°, θ_min_ = 2.3°
φ and ω scans	*h* = −22→22
Absorption correction: multi-scan (SADABS; Krause et al., 2015)	*k* = −13→13
*T*_min_ = 0.672, *T*_max_ = 0.746	*l* = −23→23
18836 measured reflections	

### Refinement

**Table:**

Refinement on *F*^2^	Hydrogen site location: mixed
Least-squares matrix: full	H atoms treated by a mixture of independent and constrained refinement
*R*[*F*^2^ > 2σ(*F*^2^)] = 0.016	*w* = 1/[σ^2^(*F*_o_^2^) + (0.0222*P*)^2^ + 1.084*P*] where *P* = (*F*_o_^2^ + 2*F*_c_^2^)/3
*wR*(*F*^2^) = 0.041	(Δ/σ)_max_ = 0.001
*S* = 1.05	Δρ_max_ = 0.54 e Å^−^^3^
6836 reflections	Δρ_min_ = −0.29 e Å^−^^3^
407 parameters	Absolute structure: Flack *x* determined using 3291 quotients [(*I*^+^)-(*I*^-^)]/[(*I*^+^)+(*I*^-^)] (Parsons et al., 2013).
3 restraints	Absolute structure parameter: −0.024 (5)
Primary atom site location: dual	

### Special details

**Table:**

Geometry. All esds (except the esd in the dihedral angle between two l.s. planes) are estimated using the full covariance matrix. The cell esds are taken into account individually in the estimation of esds in distances, angles and torsion angles; correlations between esds in cell parameters are only used when they are defined by crystal symmetry. An approximate (isotropic) treatment of cell esds is used for estimating esds involving l.s. planes.

### Fractional atomic coordinates and isotropic or equivalent isotropic displacement parameters (Å^2^)

**Table:**

	*x*	*y*	*z*	*U*_iso_*/*U*_eq_	
Rh1	0.42545 (2)	0.32086 (2)	0.63412 (2)	0.01052 (5)	
P1	0.36044 (4)	0.27657 (7)	0.50515 (3)	0.01170 (12)	
C13	0.26691 (15)	0.1786 (2)	0.47885 (13)	0.0134 (4)	
C7	0.42233 (14)	0.2032 (2)	0.45283 (13)	0.0143 (4)	
C1	0.32858 (13)	0.4316 (2)	0.45497 (12)	0.0139 (4)	
C5	0.24118 (15)	0.5609 (2)	0.35082 (14)	0.0201 (5)	
H5	0.1947	0.5676	0.3063	0.024*	
C3	0.35696 (16)	0.6616 (2)	0.44367 (15)	0.0203 (5)	
H3	0.3899	0.7365	0.4621	0.024*	
C2	0.37622 (14)	0.5425 (2)	0.48234 (13)	0.0164 (4)	
H2	0.4220	0.5367	0.5276	0.020*	
C4	0.28943 (17)	0.6700 (2)	0.37831 (15)	0.0216 (5)	
H4	0.2760	0.7511	0.3521	0.026*	
C6	0.26108 (14)	0.4415 (2)	0.38862 (13)	0.0166 (4)	
H6	0.2286	0.3665	0.3692	0.020*	
C12	0.45805 (14)	0.0810 (2)	0.47564 (13)	0.0171 (4)	
H12	0.4470	0.0344	0.5155	0.021*	
C8	0.43965 (15)	0.2706 (2)	0.39424 (13)	0.0177 (5)	
H8	0.4159	0.3540	0.3786	0.021*	
C11	0.50971 (15)	0.0276 (2)	0.44006 (14)	0.0201 (5)	
H11	0.5334	−0.0560	0.4553	0.024*	
C10	0.52700 (15)	0.0957 (3)	0.38225 (14)	0.0226 (5)	
H10	0.5633	0.0597	0.3588	0.027*	
C9	0.49115 (15)	0.2166 (3)	0.35885 (14)	0.0220 (5)	
H9	0.5019	0.2624	0.3185	0.026*	
C16	0.12602 (15)	0.0287 (2)	0.45403 (14)	0.0207 (5)	
H16	0.0784	−0.0224	0.4463	0.025*	
C17	0.12845 (15)	0.1572 (2)	0.47878 (14)	0.0192 (5)	
H17	0.0824	0.1943	0.4877	0.023*	
C14	0.26341 (14)	0.0492 (2)	0.45292 (13)	0.0182 (5)	
H14	0.3092	0.0119	0.4436	0.022*	
C15	0.19314 (15)	−0.0249 (2)	0.44067 (14)	0.0219 (5)	
H15	0.1913	−0.1126	0.4231	0.026*	
C18	0.19802 (15)	0.2314 (2)	0.49049 (13)	0.0162 (5)	
H18	0.1988	0.3198	0.5067	0.019*	
C19	0.31402 (18)	0.3416 (3)	0.64607 (16)	0.0123 (5)	
N2	0.26421 (12)	0.4462 (2)	0.63903 (12)	0.0146 (4)	
N1	0.27282 (11)	0.24015 (19)	0.66333 (11)	0.0139 (4)	
C20	0.19801 (14)	0.2803 (2)	0.66586 (13)	0.0174 (4)	
H20	0.1582	0.2270	0.6763	0.021*	
C21	0.19237 (13)	0.4093 (2)	0.65064 (13)	0.0161 (4)	
H21	0.1478	0.4646	0.6483	0.019*	
C31	0.48989 (13)	0.4414 (2)	0.73577 (12)	0.0154 (4)	
H31	0.4558	0.5126	0.7465	0.018*	
C30	0.47546 (17)	0.3193 (2)	0.76080 (15)	0.0142 (5)	
H30	0.4329	0.3191	0.7862	0.017*	
C27	0.54248 (14)	0.2242 (2)	0.64930 (13)	0.0162 (4)	
H27	0.5386	0.1482	0.6143	0.019*	
C34	0.54342 (18)	0.3440 (3)	0.61543 (18)	0.0166 (6)	
H34	0.5402	0.3386	0.5606	0.020*	
C28	0.58720 (15)	0.1936 (3)	0.73303 (14)	0.0196 (5)	
C29	0.53265 (16)	0.2061 (3)	0.78260 (14)	0.0189 (5)	
H29A	0.5008	0.1245	0.7784	0.023*	
H29B	0.5672	0.2160	0.8367	0.023*	
C32	0.57237 (15)	0.4875 (3)	0.73428 (14)	0.0198 (5)	
C33	0.58371 (16)	0.4668 (3)	0.65570 (16)	0.0213 (6)	
H33A	0.5610	0.5432	0.6227	0.026*	
H33B	0.6426	0.4626	0.6630	0.026*	
C22	0.30159 (15)	0.1041 (2)	0.67200 (13)	0.0169 (5)	
H22A	0.3530	0.0991	0.6603	0.020*	
H22B	0.2610	0.0487	0.6345	0.020*	
C24	0.28426 (14)	0.5819 (2)	0.62459 (13)	0.0156 (4)	
H24	0.3375	0.5809	0.6150	0.019*	
C23	0.31568 (15)	0.0504 (2)	0.75187 (14)	0.0207 (5)	
H23A	0.3354	−0.0399	0.7547	0.031*	
H23B	0.2645	0.0522	0.7632	0.031*	
H23C	0.3562	0.1043	0.7893	0.031*	
C26	0.22038 (16)	0.6381 (3)	0.55409 (15)	0.0232 (5)	
H26A	0.2152	0.5815	0.5100	0.035*	
H26B	0.2368	0.7260	0.5439	0.035*	
H26C	0.1680	0.6429	0.5630	0.035*	
C25	0.29387 (17)	0.6662 (2)	0.69523 (16)	0.0241 (5)	
H25A	0.2414	0.6733	0.7038	0.036*	
H25B	0.3127	0.7537	0.6873	0.036*	
H25C	0.3337	0.6258	0.7399	0.036*	
B1	0.47942 (19)	0.8319 (3)	0.63945 (18)	0.0216 (6)	
F1	0.50775 (10)	0.81699 (16)	0.57787 (10)	0.0298 (4)	
F2	0.45538 (12)	0.71358 (17)	0.65886 (12)	0.0393 (4)	
F3	0.41466 (19)	0.9168 (2)	0.61969 (17)	0.0748 (10)	
F4	0.54203 (14)	0.8787 (3)	0.70204 (13)	0.0667 (8)	
H28A	0.6405 (16)	0.253 (3)	0.7537 (14)	0.004 (6)*	
H32A	0.618 (2)	0.446 (3)	0.7789 (19)	0.027 (8)*	
H32B	0.5747 (19)	0.577 (3)	0.7442 (17)	0.024 (8)*	
H28B	0.605 (2)	0.102 (4)	0.736 (2)	0.039 (10)*	

### Atomic displacement parameters (Å^2^)

**Table:**

	*U*^11^	*U*^22^	*U*^33^	*U*^12^	*U*^13^	*U*^23^
Rh1	0.00993 (7)	0.01155 (8)	0.00951 (7)	−0.00034 (8)	0.00241 (5)	−0.00038 (7)
P1	0.0128 (3)	0.0112 (3)	0.0105 (3)	0.0005 (2)	0.0031 (2)	−0.0004 (2)
C13	0.0128 (11)	0.0147 (11)	0.0111 (10)	−0.0018 (8)	0.0017 (8)	0.0006 (8)
C7	0.0111 (10)	0.0183 (11)	0.0129 (10)	−0.0018 (8)	0.0030 (8)	−0.0042 (8)
C1	0.0148 (10)	0.0152 (11)	0.0119 (10)	0.0024 (8)	0.0044 (8)	0.0019 (8)
C5	0.0215 (12)	0.0231 (12)	0.0136 (10)	0.0027 (10)	0.0031 (9)	0.0035 (9)
C3	0.0227 (12)	0.0174 (12)	0.0216 (12)	−0.0043 (9)	0.0082 (10)	0.0001 (9)
C2	0.0174 (11)	0.0170 (11)	0.0143 (10)	−0.0005 (9)	0.0044 (8)	−0.0008 (9)
C4	0.0279 (13)	0.0174 (12)	0.0209 (12)	0.0036 (9)	0.0099 (11)	0.0062 (9)
C6	0.0175 (11)	0.0175 (11)	0.0134 (10)	−0.0010 (9)	0.0033 (9)	−0.0007 (8)
C12	0.0179 (11)	0.0172 (11)	0.0149 (10)	−0.0002 (9)	0.0035 (9)	−0.0030 (8)
C8	0.0193 (11)	0.0177 (12)	0.0147 (11)	−0.0021 (9)	0.0037 (9)	−0.0013 (9)
C11	0.0179 (11)	0.0196 (12)	0.0214 (11)	0.0015 (9)	0.0046 (9)	−0.0051 (9)
C10	0.0200 (12)	0.0263 (13)	0.0233 (12)	−0.0025 (10)	0.0097 (10)	−0.0106 (10)
C9	0.0240 (13)	0.0255 (13)	0.0189 (12)	−0.0060 (10)	0.0104 (10)	−0.0033 (10)
C16	0.0186 (11)	0.0222 (12)	0.0190 (11)	−0.0084 (10)	0.0029 (9)	0.0015 (9)
C17	0.0156 (11)	0.0249 (12)	0.0161 (11)	−0.0014 (9)	0.0038 (9)	0.0009 (9)
C14	0.0199 (11)	0.0154 (11)	0.0189 (11)	−0.0006 (9)	0.0058 (9)	−0.0019 (9)
C15	0.0246 (12)	0.0162 (12)	0.0221 (12)	−0.0045 (10)	0.0039 (10)	−0.0024 (9)
C18	0.0189 (12)	0.0158 (11)	0.0125 (11)	−0.0011 (9)	0.0033 (9)	−0.0021 (9)
C19	0.0122 (12)	0.0131 (12)	0.0095 (11)	−0.0012 (10)	0.0008 (10)	−0.0020 (9)
N2	0.0127 (9)	0.0148 (10)	0.0159 (10)	−0.0011 (7)	0.0041 (8)	−0.0029 (8)
N1	0.0137 (9)	0.0123 (9)	0.0163 (9)	−0.0011 (7)	0.0060 (7)	−0.0017 (7)
C20	0.0143 (11)	0.0218 (12)	0.0179 (11)	−0.0030 (9)	0.0078 (9)	−0.0033 (9)
C21	0.0115 (10)	0.0205 (11)	0.0171 (11)	−0.0011 (8)	0.0056 (8)	−0.0045 (9)
C31	0.0146 (10)	0.0175 (11)	0.0117 (10)	−0.0016 (8)	0.0014 (8)	−0.0035 (8)
C30	0.0154 (12)	0.0203 (14)	0.0058 (10)	−0.0019 (8)	0.0019 (9)	−0.0013 (8)
C27	0.0120 (10)	0.0198 (12)	0.0163 (11)	0.0038 (9)	0.0040 (9)	−0.0026 (9)
C34	0.0097 (13)	0.0261 (14)	0.0147 (13)	−0.0019 (11)	0.0051 (10)	−0.0033 (11)
C28	0.0155 (12)	0.0234 (13)	0.0167 (12)	0.0063 (9)	0.0011 (9)	0.0004 (9)
C29	0.0196 (12)	0.0213 (12)	0.0122 (10)	0.0036 (9)	0.0003 (9)	0.0039 (9)
C32	0.0173 (11)	0.0218 (13)	0.0184 (11)	−0.0061 (9)	0.0034 (9)	−0.0034 (9)
C33	0.0145 (11)	0.0256 (14)	0.0228 (13)	−0.0082 (10)	0.0048 (10)	−0.0012 (10)
C22	0.0228 (12)	0.0100 (10)	0.0205 (11)	−0.0001 (9)	0.0107 (9)	−0.0006 (8)
C24	0.0166 (11)	0.0126 (10)	0.0176 (11)	0.0005 (8)	0.0057 (9)	−0.0005 (8)
C23	0.0237 (12)	0.0165 (11)	0.0219 (12)	−0.0013 (9)	0.0076 (10)	0.0004 (9)
C26	0.0229 (12)	0.0224 (12)	0.0240 (12)	0.0059 (10)	0.0071 (10)	0.0052 (10)
C25	0.0265 (13)	0.0206 (13)	0.0271 (13)	−0.0041 (10)	0.0114 (11)	−0.0088 (10)
B1	0.0235 (14)	0.0155 (13)	0.0303 (15)	−0.0024 (10)	0.0149 (12)	0.0009 (10)
F1	0.0230 (8)	0.0435 (10)	0.0252 (8)	0.0038 (6)	0.0111 (7)	0.0005 (6)
F2	0.0433 (10)	0.0233 (8)	0.0547 (12)	−0.0095 (7)	0.0207 (9)	0.0074 (8)
F3	0.0900 (19)	0.0630 (13)	0.102 (2)	0.0594 (15)	0.0729 (19)	0.0508 (15)
F4	0.0626 (14)	0.1002 (19)	0.0541 (13)	−0.0596 (14)	0.0418 (12)	−0.0510 (13)

### Geometric parameters (Å, º)

**Table:**

Rh1—P1	2.3265 (6)	N2—C24	1.476 (3)
Rh1—C19	2.035 (3)	N1—C20	1.382 (3)
Rh1—C31	2.221 (2)	N1—C22	1.468 (3)
Rh1—C30	2.218 (3)	C20—H20	0.9500
Rh1—C27	2.198 (2)	C20—C21	1.345 (4)
Rh1—C34	2.206 (3)	C21—H21	0.9500
P1—C13	1.837 (3)	C31—H31	1.0000
P1—C7	1.830 (2)	C31—C30	1.383 (3)
P1—C1	1.828 (2)	C31—C32	1.521 (3)
C13—C14	1.401 (3)	C30—H30	1.0000
C13—C18	1.396 (3)	C30—C29	1.493 (3)
C7—C12	1.398 (3)	C27—H27	1.0000
C7—C8	1.398 (3)	C27—C34	1.377 (4)
C1—C2	1.397 (3)	C27—C28	1.521 (3)
C1—C6	1.398 (3)	C34—H34	1.0000
C5—H5	0.9500	C34—C33	1.510 (4)
C5—C4	1.389 (4)	C28—C29	1.528 (3)
C5—C6	1.392 (3)	C28—H28A	1.07 (3)
C3—H3	0.9500	C28—H28B	0.98 (4)
C3—C2	1.396 (3)	C29—H29A	0.9900
C3—C4	1.386 (4)	C29—H29B	0.9900
C2—H2	0.9500	C32—C33	1.546 (4)
C4—H4	0.9500	C32—H32A	1.03 (3)
C6—H6	0.9500	C32—H32B	0.93 (3)
C12—H12	0.9500	C33—H33A	0.9900
C12—C11	1.390 (3)	C33—H33B	0.9900
C8—H8	0.9500	C22—H22A	0.9900
C8—C9	1.387 (3)	C22—H22B	0.9900
C11—H11	0.9500	C22—C23	1.520 (3)
C11—C10	1.390 (4)	C24—H24	1.0000
C10—H10	0.9500	C24—C26	1.522 (3)
C10—C9	1.388 (4)	C24—C25	1.528 (3)
C9—H9	0.9500	C23—H23A	0.9800
C16—H16	0.9500	C23—H23B	0.9800
C16—C17	1.386 (4)	C23—H23C	0.9800
C16—C15	1.385 (4)	C26—H26A	0.9800
C17—H17	0.9500	C26—H26B	0.9800
C17—C18	1.385 (3)	C26—H26C	0.9800
C14—H14	0.9500	C25—H25A	0.9800
C14—C15	1.394 (3)	C25—H25B	0.9800
C15—H15	0.9500	C25—H25C	0.9800
C18—H18	0.9500	B1—F1	1.391 (3)
C19—N2	1.355 (4)	B1—F2	1.365 (3)
C19—N1	1.357 (3)	B1—F3	1.374 (4)
N2—C21	1.389 (3)	B1—F4	1.390 (4)
			
C19—Rh1—P1	88.37 (8)	N2—C21—H21	126.5
C19—Rh1—C31	94.87 (10)	C20—C21—N2	106.9 (2)
C19—Rh1—C30	86.75 (11)	C20—C21—H21	126.5
C19—Rh1—C27	155.53 (10)	Rh1—C31—H31	113.9
C19—Rh1—C34	167.59 (8)	C30—C31—Rh1	71.74 (14)
C31—Rh1—P1	156.62 (6)	C30—C31—H31	113.9
C30—Rh1—P1	166.84 (6)	C30—C31—C32	124.2 (2)
C30—Rh1—C31	36.30 (9)	C32—C31—Rh1	111.97 (15)
C27—Rh1—P1	99.34 (6)	C32—C31—H31	113.9
C27—Rh1—C31	87.21 (9)	Rh1—C30—H30	113.8
C27—Rh1—C30	80.55 (9)	C31—C30—Rh1	71.96 (14)
C27—Rh1—C34	36.45 (10)	C31—C30—H30	113.8
C34—Rh1—P1	91.40 (8)	C31—C30—C29	127.7 (2)
C34—Rh1—C31	80.55 (10)	C29—C30—Rh1	106.70 (16)
C34—Rh1—C30	95.97 (11)	C29—C30—H30	113.8
C13—P1—Rh1	117.95 (8)	Rh1—C27—H27	113.8
C7—P1—Rh1	116.76 (8)	C34—C27—Rh1	72.06 (16)
C7—P1—C13	105.19 (11)	C34—C27—H27	113.8
C1—P1—Rh1	108.50 (8)	C34—C27—C28	124.6 (2)
C1—P1—C13	104.04 (11)	C28—C27—Rh1	111.44 (15)
C1—P1—C7	102.66 (11)	C28—C27—H27	113.8
C14—C13—P1	122.70 (19)	Rh1—C34—H34	114.1
C18—C13—P1	118.76 (17)	C27—C34—Rh1	71.49 (15)
C18—C13—C14	118.4 (2)	C27—C34—H34	114.1
C12—C7—P1	119.16 (17)	C27—C34—C33	125.9 (3)
C12—C7—C8	119.1 (2)	C33—C34—Rh1	108.50 (19)
C8—C7—P1	121.59 (18)	C33—C34—H34	114.1
C2—C1—P1	118.23 (17)	C27—C28—C29	112.6 (2)
C2—C1—C6	119.2 (2)	C27—C28—H28A	110.0 (14)
C6—C1—P1	122.49 (18)	C27—C28—H28B	108 (2)
C4—C5—H5	120.1	C29—C28—H28A	112.1 (14)
C4—C5—C6	119.9 (2)	C29—C28—H28B	107 (2)
C6—C5—H5	120.1	H28A—C28—H28B	107 (3)
C2—C3—H3	120.2	C30—C29—C28	113.2 (2)
C4—C3—H3	120.2	C30—C29—H29A	108.9
C4—C3—C2	119.6 (2)	C30—C29—H29B	108.9
C1—C2—H2	119.8	C28—C29—H29A	108.9
C3—C2—C1	120.4 (2)	C28—C29—H29B	108.9
C3—C2—H2	119.8	H29A—C29—H29B	107.8
C5—C4—H4	119.7	C31—C32—C33	112.8 (2)
C3—C4—C5	120.6 (2)	C31—C32—H32A	109.8 (17)
C3—C4—H4	119.7	C31—C32—H32B	106.3 (19)
C1—C6—H6	119.9	C33—C32—H32A	113.6 (17)
C5—C6—C1	120.3 (2)	C33—C32—H32B	108.0 (19)
C5—C6—H6	119.9	H32A—C32—H32B	106 (3)
C7—C12—H12	120.0	C34—C33—C32	113.4 (2)
C11—C12—C7	120.1 (2)	C34—C33—H33A	108.9
C11—C12—H12	120.0	C34—C33—H33B	108.9
C7—C8—H8	119.7	C32—C33—H33A	108.9
C9—C8—C7	120.6 (2)	C32—C33—H33B	108.9
C9—C8—H8	119.7	H33A—C33—H33B	107.7
C12—C11—H11	119.8	N1—C22—H22A	109.0
C12—C11—C10	120.4 (2)	N1—C22—H22B	109.0
C10—C11—H11	119.8	N1—C22—C23	112.86 (19)
C11—C10—H10	120.1	H22A—C22—H22B	107.8
C9—C10—C11	119.8 (2)	C23—C22—H22A	109.0
C9—C10—H10	120.1	C23—C22—H22B	109.0
C8—C9—C10	120.1 (2)	N2—C24—H24	108.1
C8—C9—H9	120.0	N2—C24—C26	111.1 (2)
C10—C9—H9	120.0	N2—C24—C25	109.91 (19)
C17—C16—H16	120.1	C26—C24—H24	108.1
C15—C16—H16	120.1	C26—C24—C25	111.4 (2)
C15—C16—C17	119.9 (2)	C25—C24—H24	108.1
C16—C17—H17	120.0	C22—C23—H23A	109.5
C18—C17—C16	120.0 (2)	C22—C23—H23B	109.5
C18—C17—H17	120.0	C22—C23—H23C	109.5
C13—C14—H14	119.8	H23A—C23—H23B	109.5
C15—C14—C13	120.4 (2)	H23A—C23—H23C	109.5
C15—C14—H14	119.8	H23B—C23—H23C	109.5
C16—C15—C14	120.2 (2)	C24—C26—H26A	109.5
C16—C15—H15	119.9	C24—C26—H26B	109.5
C14—C15—H15	119.9	C24—C26—H26C	109.5
C13—C18—H18	119.5	H26A—C26—H26B	109.5
C17—C18—C13	121.1 (2)	H26A—C26—H26C	109.5
C17—C18—H18	119.5	H26B—C26—H26C	109.5
N2—C19—Rh1	132.4 (2)	C24—C25—H25A	109.5
N2—C19—N1	104.7 (2)	C24—C25—H25B	109.5
N1—C19—Rh1	122.86 (19)	C24—C25—H25C	109.5
C19—N2—C21	110.6 (2)	H25A—C25—H25B	109.5
C19—N2—C24	125.2 (2)	H25A—C25—H25C	109.5
C21—N2—C24	124.2 (2)	H25B—C25—H25C	109.5
C19—N1—C20	111.0 (2)	F2—B1—F1	109.9 (2)
C19—N1—C22	124.1 (2)	F2—B1—F3	109.5 (2)
C20—N1—C22	124.7 (2)	F2—B1—F4	108.1 (3)
N1—C20—H20	126.6	F3—B1—F1	109.4 (2)
C21—C20—N1	106.8 (2)	F3—B1—F4	110.8 (3)
C21—C20—H20	126.6	F4—B1—F1	109.2 (2)
			
Rh1—P1—C13—C14	−108.34 (19)	C4—C5—C6—C1	1.2 (4)
Rh1—P1—C13—C18	66.7 (2)	C4—C3—C2—C1	0.9 (4)
Rh1—P1—C7—C12	61.3 (2)	C6—C1—C2—C3	−0.4 (3)
Rh1—P1—C7—C8	−113.91 (18)	C6—C5—C4—C3	−0.7 (4)
Rh1—P1—C1—C2	31.68 (19)	C12—C7—C8—C9	0.4 (3)
Rh1—P1—C1—C6	−151.50 (17)	C12—C11—C10—C9	−1.3 (4)
Rh1—C19—N2—C21	178.1 (2)	C8—C7—C12—C11	−0.3 (3)
Rh1—C19—N2—C24	−4.5 (4)	C11—C10—C9—C8	1.5 (4)
Rh1—C19—N1—C20	−178.26 (17)	C16—C17—C18—C13	−0.9 (4)
Rh1—C19—N1—C22	−2.8 (3)	C17—C16—C15—C14	0.8 (4)
Rh1—C31—C30—C29	97.5 (3)	C14—C13—C18—C17	1.7 (3)
Rh1—C31—C32—C33	12.8 (3)	C15—C16—C17—C18	−0.3 (4)
Rh1—C30—C29—C28	43.2 (2)	C18—C13—C14—C15	−1.3 (3)
Rh1—C27—C34—C33	99.8 (3)	C19—N2—C21—C20	0.6 (3)
Rh1—C27—C28—C29	13.9 (3)	C19—N2—C24—C26	124.9 (2)
Rh1—C34—C33—C32	39.5 (3)	C19—N2—C24—C25	−111.3 (3)
P1—C13—C14—C15	173.84 (18)	C19—N1—C20—C21	−0.6 (3)
P1—C13—C18—C17	−173.57 (18)	C19—N1—C22—C23	118.0 (3)
P1—C7—C12—C11	−175.64 (18)	N2—C19—N1—C20	0.9 (3)
P1—C7—C8—C9	175.65 (19)	N2—C19—N1—C22	176.4 (2)
P1—C1—C2—C3	176.55 (18)	N1—C19—N2—C21	−0.9 (3)
P1—C1—C6—C5	−177.46 (18)	N1—C19—N2—C24	176.4 (2)
C13—P1—C7—C12	−71.6 (2)	N1—C20—C21—N2	0.0 (3)
C13—P1—C7—C8	113.2 (2)	C20—N1—C22—C23	−67.2 (3)
C13—P1—C1—C2	158.06 (18)	C21—N2—C24—C26	−58.0 (3)
C13—P1—C1—C6	−25.1 (2)	C21—N2—C24—C25	65.8 (3)
C13—C14—C15—C16	0.0 (4)	C31—C30—C29—C28	−36.6 (4)
C7—P1—C13—C14	23.9 (2)	C31—C32—C33—C34	−35.3 (3)
C7—P1—C13—C18	−161.03 (19)	C30—C31—C32—C33	95.1 (3)
C7—P1—C1—C2	−92.49 (19)	C27—C34—C33—C32	−40.7 (4)
C7—P1—C1—C6	84.3 (2)	C27—C28—C29—C30	−39.1 (3)
C7—C12—C11—C10	0.7 (4)	C34—C27—C28—C29	96.4 (3)
C7—C8—C9—C10	−1.0 (4)	C28—C27—C34—Rh1	−104.1 (2)
C1—P1—C13—C14	131.5 (2)	C28—C27—C34—C33	−4.3 (4)
C1—P1—C13—C18	−53.5 (2)	C32—C31—C30—Rh1	−104.6 (2)
C1—P1—C7—C12	179.85 (18)	C32—C31—C30—C29	−7.1 (4)
C1—P1—C7—C8	4.6 (2)	C22—N1—C20—C21	−176.0 (2)
C2—C1—C6—C5	−0.7 (3)	C24—N2—C21—C20	−176.8 (2)
C2—C3—C4—C5	−0.4 (4)		

### Hydrogen-bond geometry (Å, º)

**Table:**

*D*—H···*A*	*D*—H	H···*A*	*D*···*A*	*D*—H···*A*
C12—H12···F1^i^	0.95	2.57	3.245 (3)	129
C21—H21···F4^ii^	0.95	2.52	3.082 (3)	118
C22—H22*A*···F3^i^	0.99	2.39	3.120 (3)	130
C24—H24···F2	1.00	2.37	3.139 (3)	133
C32—H32*B*···F2	0.93 (3)	2.57 (3)	3.094 (3)	116 (2)
C28—H28*B*···F4^i^	0.98 (4)	2.53 (4)	3.318 (4)	138 (3)

Symmetry codes: (i) *x*, *y*−1, *z*; (ii) *x*−1/2, *y*−1/2, *z*.
